# Epidemiological and Clinical Characteristics and Risk Factors for Death of Patients with Avian Influenza A H7N9 Virus Infection from Jiangsu Province, Eastern China

**DOI:** 10.1371/journal.pone.0089581

**Published:** 2014-03-04

**Authors:** Hong Ji, Qin Gu, Li-ling Chen, Ke Xu, Xia Ling, Chang-jun Bao, Fen-yang Tang, Xian Qi, Ying-qiu Wu, Jing Ai, Gu-yu Shen, Dan-jiang Dong, Hui-yan Yu, Mao Huang, Quan Cao, Ying Xu, Wei Zhao, Yang-ting Xu, Yu Xia, Shan-hui Chen, Gen-lin Yang, Cai-ling Gu, Guo-xiang Xie, Ye-fei Zhu, Feng-cai Zhu, Ming-hao Zhou

**Affiliations:** 1 Department of Acute Infectious Disease Control and Prevention, Jiangsu Provincial Center for Disease Control and Prevention, Nanjing, Jiangsu, China; 2 Department of Critical Care Medicine, Nanjing Drum Tower Hospital, Nanjing, Jiangsu, China; 3 Suzhou Municipal Center for Disease Control and Prevention, Suzhou, Jiangsu, China; 4 Wuxi Municipal Center for Disease Control and Prevention, Wuxi, Jiangsu, China; 5 The First Affiliated Hospital of Soochow University, Suzhou, Jiangsu, China; 6 Wuxi People's Hospital Affiliated to Nanjing Medical University, Wuxi, Jiangsu, China; 7 The First Affiliated Hospital with Nanjing Medical University, Nanjing, Jiangsu, China; 8 The Second Hospital of Nanjing, Nanjing, Jiangsu, China; 9 Nanjing Municipal Center for Disease Control and Prevention, Nanjing, Jiangsu, China; 10 Jiangsu Provincial Center for Disease Control and Prevention, Nanjing, Jiangsu, China; University of Minnesota, United States of America

## Abstract

**Background:**

A novel avian influenza A (H7N9) virus has caused great morbidity as well as mortality since its emergence in Eastern China in February 2013. However, the possible risk factors for death are not yet fully known.

**Methods and Findings:**

Patients with H7N9 virus infection between March 1 and August 14, 2013 in Jiangsu province were enrolled. Data were collected with a standard form. Mean or percentage was used to describe the features, and Fisher's exact test or *t*-test test was used to compare the differences between fatal and nonfatal cases with H7N9 virus infection. A total of 28 patients with H7N9 virus infection were identified among whom, nine (32.1%) died. The median age of fatal cases was significant higher than nonfatal cases (*P*<0.05). Patients with older age were more strongly associated with increased odds of death (OR = 30.0; 95% CI, 2.85–315.62). Co-morbidity with chronic lung disease and hypertension were risk factors for mortality (OR = 14.40; 95% CI, 1.30–159.52, OR = 6.67; 95% CI, 1.09–40.43, respectively). Moreover, the presence of either bilateral lung inflammation or pulmonary consolidation on chest imaging on admission was related with fatal outcome (OR = 7.00; 95%CI, 1.10–44.61). Finally, dynamic monitoring showed that lymphopenia was more significant in fatal group than in nonfatal group from day 11 to week five (*P*<0.05). The decrease in oxygenation indexes were observed in most cases and more significantly in fatal cases after week three (*P*<0.05), and the value of nearly all fatal cases were below 200 mmHg during our evaluation period.

**Conclusions:**

Among cases with H7N9 virus infection, increased age accompanied by co-morbidities was the risk of death. The severity of lung infection at admission, the persistence of lymphocytopenia, and the extended duration of lower oxygenation index all contributed to worsened outcomes of patients with H7N9 virus infection.

## Introduction

Avian influenza (AI) is an infectious disease in birds caused by type A strains of influenza virus. Some of these AI viruses have also been reported to cross the species barrier and caused disease or subclinical infections in humans and other mammals [1]. H5, H7 and H9 subtypes have been reported to cause human infections sporadically. With the exception of H5 subtype, which had resulted in more than 600 human cases with an over 60% mortality worldwide [2], H7 and H9 subtypes often led to mild to moderate infections and only one fatal case caused by H7N7 reported in Netherland [3].

Since the emergence of human infection with H7N9 virus in February 2013, much effort has been devoted to rapidly understanding the epidemiology, severity, and impact of this novel influenza virus [4]–[8]. As of 25 October 2013,137 human infections had been confirmed, with 45 deaths [9]. Fatalities associated with H7N9 virus infection were of great public health concern. However, only limited data addressed this point [10]. In order to better describe the epidemiological and clinical features, the progression of this disease, and the risk factors associated with fatal outcome, we retrospectively and prospectively reviewed medical records of 28 laboratory-confirmed cases of H7N9 virus infection in Jiangsu Province, Eastern China, between March 1 and August 14, 2013.

## Methods

### Enrollment of patients

Case definitions were established based on “The diagnosis and treatment programs of human infections with H7N9 virus” issued by the National Health and Family Planning Commission of the People's Republic of China [11]. Patients with laboratory-confirmed infection in Jiangsu Province were enrolled. The laboratory test assays for H7N9 virus that we performed have been described previously [12]. The patients were classified into fatal and nonfatal group according to their ultimate outcomes.

### Data collection

A standard questionnaire was used to collect information about demographic and epidemiologic characteristics, the dates of illness onset, visiting clinical facilities, hospital admission, and clinical outcomes (discharge or death), symptoms, chest radiography or computed tomography (CT) findings on admission, oseltamivir treatment and dynamic laboratory testing including white blood counts (WBC), lymphocytes, platelet (PLT), aspartate aminotransferase (AST), alanine aminotransferase (ALT), serum creatinine (SCr), and oxygenation indexes (PaO2/FiO2) since disease onset. Data were retrospectively collected by reviewing medical records and interviewing relatives, contacts, and health workers who provided medical care for the patients before hospitalization. Among the patients chosen, 25 patients were followed and observed between March 20, 2013, and May 10, 2013 after hospitalization. Their vital dynamic laboratory findings were prospectively monitored and collected. These data were reviewed by a trained team of physicians and medical students, and entered in duplicate into an electronic database.

Before investigation, patients or their guardians were informed of the purpose of the study by trained investigators. Oral consent was obtained from patients who were illiterate. A literate witness was available to sign on behalf of the participant after the patient had given oral consent, and the ethics committee of the Jiangsu Provincial Center for Disease Control and Prevention gave approval for the verbal consent procedure. Written informed consent for the use of the epidemiological and clinical data was obtained from all patients or their guardians on their behalf. This study was approved by the ethics committee of JSCDC.

### Statistical analysis

Vital signs and laboratory values were dichotomized into normal or abnormal on the basis of normal ranges. Continuous variables were summarized in average and standard variance which followed normal distribution or the median (with interquartile ranges) for those were non-normally distributed. For categorical variables, the percentages of patients in each category were calculated. For bivariate analysis, Chi-square or Fisher's exact test was used to compare categorical outcomes, and common odds ratios (ORs) and confidence intervals (CIs) were calculated. The independent-samples *t*-test and Wilcoxon rank-sum test were used for comparison of two groups with continuous outcome. The Kaplan-Meier product-limit method was used to analyze patient survival fraction. Patients who were discharged and critically ill were assumed to survive. The log-rank test was used to compare survival distributions. Hypothesis testing was conducted using a two-sided test, with an alpha value of 0.05 to indicate statistical significance. SPSS 17.0 (SPSS, Chicago, Illinois) and GraphPad Prism® version 5.01 were used to analyze the results.

## Results

### Epidemiological and clinical characteristics of cases with H7N9 virus infection

A total of 28 laboratory confirmed human cases with H7N9 virus infection had been reported in Jiangsu Province, Eastern China by August 14, 2013. Aside from one case, other 27 (96.4%) cases needed hospitalization. Among the 27, 24 (85.7%) cases were subsequently transferred to the Intensive Care Unit (ICU) and nine (32.1%) cases died. The onset of the first case was on March 8, and most of the cases were aggregated in the period from late March to early April (Figure S1). The average age of all cases was 52.9 years old (range, 15 to 85), and the proportion of male cases was over 70% (20/28). Notably, over 85% (24/28) cases came from urban areas. Cases were from nine out of 13 municipal prefectures in Jiangsu Province. However, most cases came from Nanjing (12), the capital of Jiangsu Province, and Suzhou (6), which was adjacent to both Shanghai and Zhejiang Province (Figure S2).

Data on exposure to animals 14 days before illness onset were available from 19 of the 28 patients. Among them, 11 (39.3%) reported a definite history of recent exposure to animals, and 8 (28.6%) reported a possible history of recent exposure to animals. Ten cases (52.6%) were exposed to chickens, and 6 cases (31.6%) to pigeons. Other animals included quail, wild birds, pet birds, and pigs. On the basis of data from the 11 patients who provided a definite date of exposure to live poultry, we estimated the median incubation period to be 6.0 days (range, 1 to 7). Nearly all (96%, 27/28) patients with H7N9 virus infection received treatment with oseltamivir. However, the median time from illness onset to first administration was 7 days (range 1 to 16 days) (Table 1).

**Table 1:** Epidemiological characteristic of fatal (n = 9) and nonfatal (n = 19) cases with novel avian influenza A(H7N9) virus infections, Jiangsu province, Eastern China, 2013.

Characteristics	Total	Fatal case	Nonfatal case	*P*	Unadjusted OR (95%CI)
Male sex*	71.4(20/28)	77.8(7/9)	68.4(13/19)	1.00^c^	1.62(0.26–10.23)
Age (yrs.)					
Mean ± SD	52.9±20.4	68.8±16.5	45.4±17.8	0.00^b^	NA
Subgroup (yrs.)*					
15–39	28.6(8/28)	11.1(1/9)	36.8(7/19)	0.21^c^	0.31(0.02–2.09)
40–59	28.6(8/28)	0.0(0/9)	29.2(8/19)	0.03^c^	1.73(1.18–2.54)
60+	42.9(12/28)	88.9(8/9)	21.1(4/19)	0.00^c^	30.0(2.85–315.62)
Type of residence*					
Rural	14.3(4/28)	11.1(1/9)	15.8(3/19)	1.00^c^	0.67(0.06–7.48)
Urban	85.7(24/28)	88.9(8/9)	84.2(16/19)	1.00^c^	1.50(0.13–16.82)
Co-morbidities*					
Chronic lung disease	17.9(5/28)	44.4(4/9)	5.3(1/19)	0.03^c^	14.40(1.30–159.52)
Hypertension	28.6(8/28)	55.6(5/9)	15.8(3/19)	0.04^c^	6.67(1.09–40.43)
Diabetes	14.3(4/28)	22.2(2/9)	10.5(2/19)	0.57^c^	2.43(0.28–20.82)
Cancer	7.1(2/28)	11.1(1/9)	5.3(1/19)	1.00^c^	2.25(0.13–40.66)
Coronary heart disease	10.7(3/28)	11.1(1/9)	10.5(2/19)	1.00^c^	1.06(0.08–13.52)
Rheumatoid arthritis	7.1(2/28)	0.0(0/9)	10.5(2/19)	1.00^c^	0.90(0.77–1.05)
Hepatitis B infection	3.6(1/28)	0.0(0/9)	5.3(1/19)	1.00^c^	0.95(0.85–1.05)
Cerebrovascular disease	3.6(1/28)	0.0(0/9)	5.3(1/19)	1.00^c^	0.95(0.85–1.05)
Pregnancy	3.6(1/28)	0.0(0/9)	5.3(1/19)	1.00^c^	0.95(0.85–1.05)
≥1 condition	60.7(17/28)	62.5(6/9)	57.9(11/19)	1.00^c^	1.46(0.28–7.64)
Time from illness onset to first medical care(days)					
Median(IQR)	1.0(0.0–2.0)	1.0(0.5–4.0)	1.0(0.0–2.0)	0.14^a^	NA
Time from illness onset to hospitalization(days)					
Median(IQR)	4.0(3.0–6.0)	4.0(3.0–9.5)	4.5(3.8–5.0)	0.60^a^	NA
Time from illness onset to ARDS(days)					
Median(IQR)	8.0(5.0–9.3)	8.5(4.0–9.0)	7.5(5.0–11.0)	0.95^a^	NA
The period of hospitalization (days)					
Median(IQR)	33(22–52)	31.0(21.5–51.0)	34.5(22.8–61.8)	0.21^a^	NA
Type of exposure to animals*					
Definite	32.1(9/28)	33.3(3/9)	31.6(6/19)	1.00^c^	1.08(0.20–5.87)
Possible	28.6(8/28)	22.2(2/9)	31.6(6/19)	1.00^c^	0.62(0.10–3.92)
None	39.3(11/28)	44.4(5/9)	36.8(7/19)	0.43^c^	2.14(0.43–10.74)
Oseltamivir treatment	96.4(27/28)	100.0(9/9)	94.70(18/19)	1.00^c^	1.06(0.95–1.17)
Time from illness onset to oseltamivir treatment(days)					
Median(IQR)	7.0(3.8–10.0)	10(5.0–12.0)	7.0(3.5–10.0)	0.22^a^	NA
Time from hospitalization to oseltamivir treatment (days)					
Median(IQR)	2.0(0.0–4.0)	3.0(1.5–3.5)	1.0(0.0–4.5)	0.37^a^	NA

*Data are % (n/N), Fisher's exact test was used.

a Wilcoxon rank-sum test was used;

b Independent samples t test was used;

c Fisher's exact test was used.

All 28 patients presented with fever on admission. The maximum median body temperature was 39.5°C (range, 38.2°C to 41.0°C). Cough, sputum production, fatigue, myalgia and arthralgia, and chest tightness were also common manifestations in all patients with H7N9 virus infection. Other symptoms such as sore throat, headache, chill, rhinorrhea and diarrhea were comparatively less common. The primary laboratory abnormalities on admission were lymphocytopenia (81.0%), decreased serum albumin (83.3%), and elevated C-reactive protein (CRP) (90.0%), lactate dehydrogenase (LDH) (85.7%), creatine kinase (CK) (84.6%) and AST (73.3%). In addition, slight leukocytopenia (46.4%), thrombocytopenia (42.9%), decreased SCr (33.3%), elevated ALT (50.0%), blood urea nitrogen (BUN) (41.7%), and serum CKMB (37.5%) were often observed. About 48.1% of patients developed either bilateral lung inflammation or pulmonary consolidation, which was determined by chest radiography or computed tomography (CT) (Table 2).

**Table 2:** Clinical characteristics and laboratory findings on admission of fatal (n = 9) and nonfatal (n = 19) cases with novel avian influenza A (H7N9) virus infections, Jiangsu province, Eastern China, 2013.

Variables	Total	Fatal case	Nonfatal cases	*P*	Unadjusted OR(95%CI)
Symptoms*					
Fever	100.0(28/28)	100.0(9/9)	100.0(19/19)	NA	NA
The highest temperature(°C)					
Median(range)	39.5(38.2–41.0)	39.6(39.0–40.0)	39.4(38.2–41.0)	NA	NA
Cough*	85.7(24/28)	88.9(8/9)	84.2(16/19)	1.00^a^	1.50(0.13–16.82)
Sputum production*	46.4(13/28)	66.7(6/9)	36.8(7/19)	0.23^a^	3.43(0.65–18.22)
Fatigue*	39.3(11/28)	33.3(3/9)	42.1(8/19)	1.00^a^	0.69(0.13–3.61)
Chest tightness*	35.7(10/28)	33.3(3/9)	36.8(7/19)	1.00^a^	0.86(0.16–4.55)
Myalgia/Arthralgia*	32.1(9/28)	22.2(2/9)	36.8(7/19)	0.67^a^	0.49(0.08–3.05)
Shortness of breath*	28.6(8/28)	33.3(3/9)	26.3(5/19)	1.00^a^	1.40(0.25–7.83)
Dyspnea*	25.0(7/28)	22.2(2/9)	26.3(5/19)	1.00^a^	0.80(0.12–5.21)
Headache*	17.9(5/28)	22.2(2/9)	15.8(3/19)	1.00^a^	1.52(0.21–11.23)
Sore throat*	17.9(5/28)	11.1(1/9)	21.1(4/19)	1.00^a^	0.47(0.05–4.93)
Rhinorrhea*	14.3(4/28)	11.1(1/9)	15.8(3/19)	1.00^a^	0.67(0.06–7.48)
Nausea/vomiting*	14.3(4/28)	22.2(2/9)	10.5(2/19)	0.57^a^	2.43(0.28–20.82)
Chill*	7.1(2/28)	22.2(2/9)	0.0(0/19)	0.10^a^	0.78(0.55–1.16)
Diarrhea*	3.6(1/28)	0.0(0/9)	5.3(1/19)	1.00^a^	1.06(0.95–1.17)
WBC(×10^9^/L)*					
<4.0	46.4(13/28)	44.4(4/9)	47.4(9/19)	1.00^a^	0.98(0.20–4.76)
>10.0	0.0(0/28)	0.0(0/9)	0.0(0/19)	NA	NA
Neutrophils counts (×10^9^/L)*					
<2.0	35.7(10/28)	44.4(4/9)	31.6(6/19)	0.68^a^	1.73(0.34–8.87)
>7.0	3.6(1/28)	0.0(0/9)	5.3(1/19)	1.00^a^	1.06(0.95–1.17)
Lymphocytescounts (×10^9^/L)*					
<0.8	81.0(17/21)	100.0(6/6)	78.6(11/15)	0.28^a^	1.36(1.01–1.85)
>4.0	0.0(0/21)	0.0(0/6)	0.0(0/15)	NA	NA
PLT (×10^9^/L)*					
<100	42.9(6/14)	50.0(3/5)	44.4(3/9)	0.58^a^	3.00(0.31–28.84)
>300	0.0(0/14)	0.0(0/5)	0.0(0/9)	NA	NA
SCr(ummol/L)*					
<62 ummol/L	33.3(5/15)	16.7(1/6)	44.4(4/9)	0.58^a^	0.25(0.02–3.10)
>106 ummol/L	6.7(1/15)	16.7(1/6)	0.0(0/9)	0.40^a^	0.83(0.58–1.19)
LDH(U/L)*					
<109 U/L	0.0(0/14)	0.0(0/5)	0.0(0/9)	NA	NA
>245 U/L	85.7(12/14)	100.0(5/5)	77.8(7/9)	0.51^a^	1.29(0.91–1.82)
ALT(U/L)*					
>40 U/L	50.0(6/12)	66.7(2/3)	44.4(4/9)	1.00^a^	2.50(0.16–38.60)
AST(U/L)*					
>40 U/L	73.3(11/15)	100.0(5/5)	60.0(6/10)	0.23^a^	1.67(1.00–2.77)
BUN(mmol/L)*					
<2.5 mmol/L	8.3(1/12)	0.0(0/4)	12.5(1/8)	1.00^a^	1.14(0.88–1.49)
>6.1 mmol/L	41.7(5/12)	75.0(3/4)	25.0(2/8)	0.22^a^	9.00(0.56–143.89)
CRP (mg/L)*					
>10.0 mg/L	90.0(9/10)	100.0(4/4)	83.3(5/6)	1.00^a^	1.20(0.84–1.72)
CK(U/L)*					
<20.0 U/L	0.0(0/13)	0.0(0/4)	0.0(0/9)	NA	NA
>185.0 U/L	84.6(11/13)	100.0(4/4)	88.9(7/9)	1.00^a^	1.29(0.91–1.83)
CK-MB isoenzyme(U/L)*					
<2.0 U/L	0.0(0/8)	0.0(0/1)	0.0(0/7)	NA	NA
>24.0 U/L	37.5(3/8)	0.0(0/1)	42.9(3/7)	1.00^a^	1.50(0.95–2.38)
Human Albumin(g/L)*					
<35 g/L	83.3(10/12)	100.0(6/6)	71.4(4/6)	0.46^a^	1.50(0.85–2.64)
>51 g/L	0.0(0/12)	0.0(0/6)	0.0(0/6)	NA	NA
Either bilateral lung inflammation or pulmonary consolidation*	48.1(13/27)	77.8(7/9)	33.3(6/18)	0.04^a^	7.00(1.10–44.61)

*Data are % (n/N), NA: not available,

a Fisher's exact test was used.

### Risk factors associated with fatal outcome due to H7N9 virus infection

**Figure 1:**
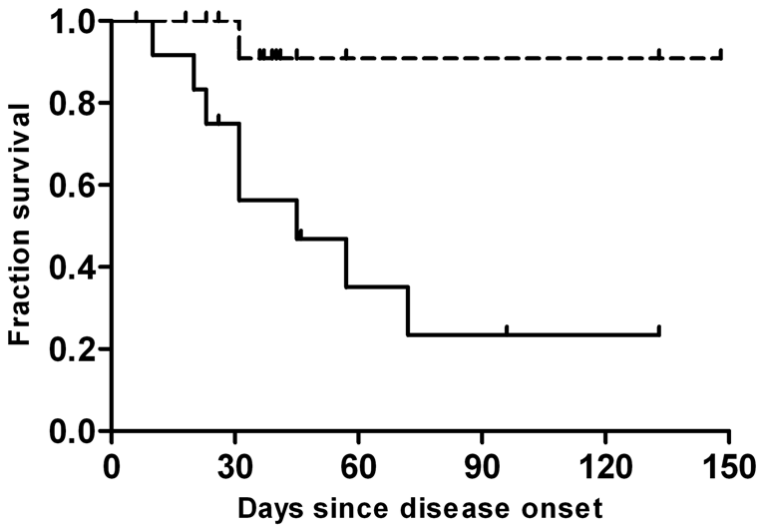
Survival plot for cases aged <60 years (dashed line), compared with cases aged ≥60 years(solid line), by duration since onset of illness.

The median age of patients with confirmed H7N9 virus infection among fatal cases was 68.8 years (range, 32 to 85), which was statistically higher than that of nonfatal cases with a median age of 45.4 years (range, 15 to 74) (*P*<0.05). The median period from disease onset to death among fatal cases was 31.0 days (range, 10 to 72), yet the median among nonfatal cases from onset to endpoint (May 10, 2013) was 39.5 days (range, 6 to 148). Furthermore, the survival curve for patients aged≥60 years, compared with patients<60 years (OR = 30.0, 95% CI, 2.85–315.62), demonstrated evidence for a survival difference between these two groups (*P*<0.001) (Figure 1). The prevalence of chronic lung diseases (mainly asthma and chronic obstructive pulmonary disease) in fatal cases and nonfatal cases were 44.4%, 5.3%, respectively, suggesting chronic lung diseases served as a risk factor for death (OR = 14.40; 95% CI, 1.30–159.52). The prevalence of hypertension among fatal cases was 55.6%, significantly higher than that of the nonfatal group (15.8%) (*P*<0.05), implying hypertension was also a risk factor for mortality (OR = 6.67; 95% CI, 1.09–40.43). Interestingly, almost of the fatal patients aged 60 years or older (7/8) had one or more co-morbidities, yet of 16 patients <60 years , 62.5% (10/16) patients had no co-morbidities, and only one patient with the age of 32 years older died. Regarding the effect of oseltamivir, no significant difference was observed despite the fact that the median day of administration in fatal group (10 days) was comparatively longer than that of the non-fatal group (7 days). All the median duration from illness onset to seek healthcare, to admission, and to the presence of acute respiratory distress syndrome (ARDS) did not vary significantly between the two groups (Table 1). Notably, the presence of either bilateral lung inflammation or pulmonary consolidation on chest radiography or CT on admission was statistically higher in fatal cases and served as an important predictor for risk of death (OR = 7.00; 95%CI, 1.10–44.61). In addition, by comparing the proportions of symptoms and laboratory parameters on admission between the fatal group and the nonfatal group, the results showed that almost all the parameters were relatively higher and more serious in fatal cases. However, no statistically significant difference was observed (*P*>0.05) (Table 2).

### Dynamic profiles of laboratory findings between fatal and nonfatal cases with H7N9 virus infection

**Figure 2:**
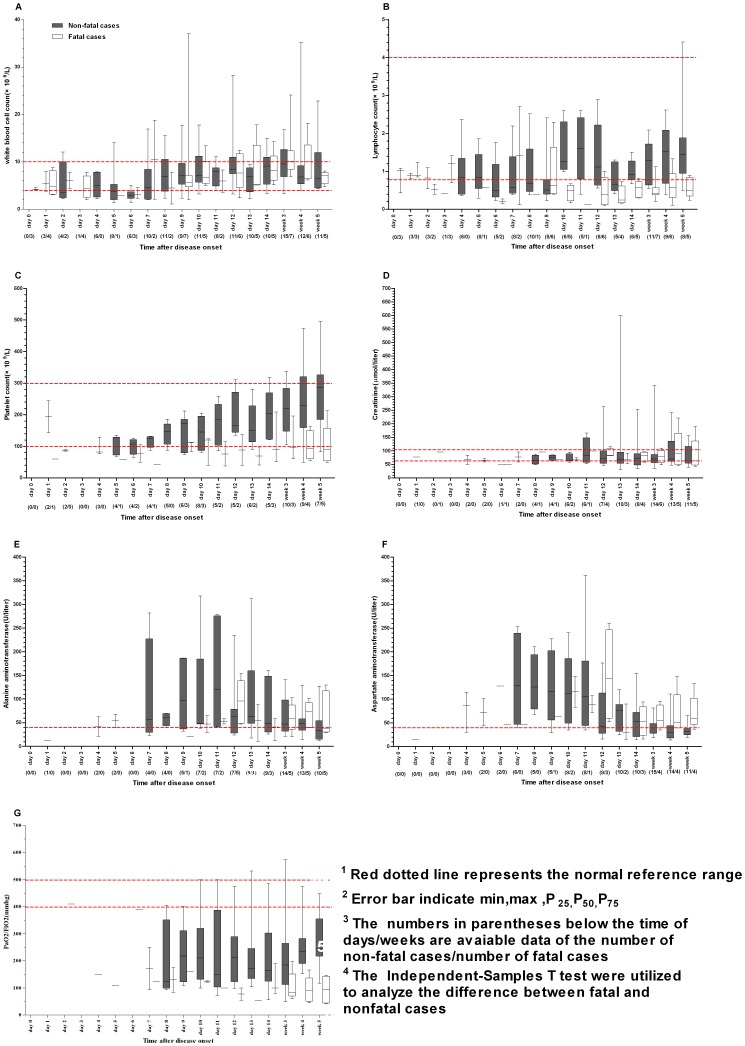
Dynamic profiles of some laboratory parameters between fatal cases and nonfatal cases with novel avian influenza A (H7N9) virus infection. A - WBC, B - lymphocyte counts, C - PLT, D - Creatinine, E – ALT, F – AST, and G - PaO2/FiO2.

In observing the dynamic changes of laboratory findings between the two groups, WBC, lymphocytes and PLT dropped to their lowest point at five to six days after disease onset (Figure 2-A, 2-B and 2-C). Thereafter, lymphocyte counts in the nonfatal group converted to the normal limit after day 10. whereas the value in the fatal group were still kept lower and showed a significant difference from day 11 to week five compared to the nonfatal group (*P*<0.05) (Figure 2-B). The median WBC in both groups gradually converted to normal range (Figure 2-A), but PLT counts almost recovered to normal limits only in the nonfatal group (Figure 2-C). However, no significant differences in WBC and PLT were found in the following days and weeks. Although acute liver injury, defined as AST or ALT>40 U/L, was common after disease onset in between groups, there was no significantly difference during the evaluation period (Figure 2-E and Figure 2-F). No statistically significant difference was observed in the median time of first appearance of pulmonary respiratory dysfunction, defined as oxygenation indexes (PaO2/FiO2) <300 mmHg, between the fatal group (day 10) and the nonfatal group (day 11). But pulmonary respiratory dysfunction was observed in most cases and was significantly more serious in fatal cases since week three (*P*<0.05) (Figure 2-G), and the value of almost all fatal cases were lower than 200 mmHg. In contrast, acute renal injury, defined as SCr>134 µmol/L, was relatively low in both groups, and no statistically significant difference was found. Most cases had SCr values within the reference range (Figure 2-D).

According to the key time points above, especially the dynamic change of lymphocyte counts during our evaluation period, we preliminary inferred the progression was divided into three phases: period of prodromal (from day three to six) when cases presented with fever and cough accompanied by abnormalities of partial laboratory parameters like WBC, lymphocyte counts and liver-related enzymes; the period of apparent manifestation (from day seven to nine) when most of cases developed ARDS: and two prognosis outcomes including fatal outcome and convalescent period (after day nine), when laboratory parameters in nonfatal cases started to convert to normal while those in fatal cases became worse (Figure S3).

## Discussion

We described the detailed epidemiological and clinical admission features of 28 laboratory confirmed H7N9 virus infections identified in Jiangsu province Eastern China, in 2013. Moreover, we identified a number of important risk factors for fatal outcome including age, co-morbidity such as chronic lung disease, and presence of either bilateral lung inflammation or pulmonary consolidation on chest radiography or CT at admission. Aside from that, through dynamic comparison of key laboratory parameters between fatal cases and nonfatal cases, we found that sustained lymphocytopenia, sustained decreasing oxygenation indexes levels below 200 mmHg would predict a poor outcome.

Our findings suggested that older patients with confirmed H7N9 virus infection were associated with increased risks for death. As describe by Cowling B.J.et al, the risk of serious illness after H7N9 infection was 5.1 times higher in persons of 65 years or older versus younger ages [13]. By contrast, H5N1 virus infection showed a pattern of a decrease in the risk of death with increase age [14]–[16]. One possible differential explanation for this pattern was that ageing was associated with a decline in the diversity of the T cells' repertoire, leading to impaired immunity to influenza [17], and cross-reactive humoral responses against H5N1 virus infection may be induced by other influenza subtypes [18]. The prevalence of chronic lung disease in fatal cases was significant higher than in nonfatal cases, and thus increased the risk of death. Asthma and other chronic respiratory diseases have long been recognized as important risk factors for severe illness from influenza infection [19], [20]. Additionally, hypertension was overrepresented amongst fatal cases compared with nonfatal cases, and also led to increased risk of death. However, it is known that both hypertension and chronic lung disease are related to age. Therefore, a high proportion of elderly patients with severe H7N9 virus infection may due to decreased immune function or to underlying chronic disease [21], and the immunological status of the elderly host with co-morbidities could increase the risk of death [22].

Critically, the patients in our series presented primarily with fever, cough, and sputum production. This clinical picture was consistent with other published season influenza virus infection [23]. H7N9 virus infection cases were more likely to have lymphocytopenia, thrombocytopenia, and elevated serum liver enzymes (AST and ALT) on admission, which were generally similar to patients with H5N1 virus infection [14], [24], [25] and pdm2009 H1N1 virus infection [23], [26]. Most clinical features at hospital admission were not useful in estimating risk factors for fatal outcomes. In contrast, neutropenia, elevated ALT, vomiting and diarrhea on admission were reported associated with fatal H5N1 virus infection outcome [14], [27], [28]. Elevated SCr levels remained more common among hospitalized patients who died than hospitalized patients who survived with pdm2009 H1N1 virus infection [26]. In particular, the presentation of either bilateral lung inflammation or pulmonary consolidation at admission among patients with H7N9 virus infection was an important predictor risk factor for death. These observations indicated the early clinical symptoms of H7N9 virus infection were usually influenza-like symptoms, but the death predictor sensitivity or specificity of any of these factors cannot, however, be assessed with our data. Efforts should be made to compare H7N9 fatal cases with nonfatal cases by severity, determined by the findings of chest radiograph and CT inspects on admission.

Most importantly, our study suggested that a decreasing lymphocyte counts was more commonly observed in fatal cases than nonfatal cases during the study period, suggesting a poor outcome after day 11. While in H5N1 report, the magnitude of the increase of neutrophil counts was greater among fatal cases from zero to eight days after hospital admission [14]. In addition, we also found pulmonary respiratory dysfunction was common in all cases and more serious in fatal cases, whereas this presentation was lower in patients infected with pdm2009 H1N1 virus as reported previously [29]. The decreasing oxygenation index below 200 mmHg was a risk factor of prognosis for critically ill patients with H7N9 virus infection. The above results may help physicians to generate clinical treatment strategies and decrease case-fatalities.

The clinical benefit of early antiviral treatment with oseltamivir has been strongly proved during pdm2009 H1N1 and H5N1 virus infections [30], [31]. National guidelines also recommend that antiviral treatment with oseltamivir should be administered as soon as possible in patients with suspected or confirmed cases of H7N9 virus infection [32]. However, in two of our study groups oseltamivir was administrated with a long time interval following onset of symptoms. The time delays which may greatly affect the effectiveness of treatment were mainly attributed to little knowledge of this new disease.

The current case-fatality rate for H7N9 virus infection was 32.1%, which was slightly higher than the previous report [4], but lower than that of H5N1 virus infection [2], and much higher than that for seasonal influenza and pdm2009 H1N1 virus infection [30]. However, the exact case-fatality rate for H7N9 virus infection still needs to be studied further, because the full clinical spectrum of H7N9 virus infection may not yet be known.

Severely ill patients were more likely to be identified in the emerging infectious diseases [33]. Measures to enhance surveillance for severe and mild human illness with H7N9 virus infection were needed to implement.

Our study is subject to several limitations. First, we did not collect sufficient information from all patients on laboratory results in the early stages, which was useful to identify and compare the difference between the fatal group and the nonfatal group. Second, the sample size was small because of limited cases, which restricted the ability of the study to uncover significant variables.

In conclusion, we have reported some important epidemiological and clinical variables between fatal and nonfatal cases of H7N9 virus infection. This report will benefit public health officers, and those clinicians who are at the frontline of recognition and report the cases. However, many questions still remain unanswered. Expanding and enhancing surveillance will help in the early discovery and diagnosis of suspected cases, which will reduce the number of severe cases and deaths.

## Supporting Information

Figure S1 Onset time distribution (by week) of cases with avian influenza A (H7N9) virus infection, Jiangsu province, Eastern China, 2013 (n = 28).
(TIF)

Figure S2 Geographical distribution of cases with a novel avian influenza A (H7N9) virus infection, Jiangsu Province, Eastern China, 2013 (n = 28).
(TIF)

Figure S3 Stochastic natural cause of disease caused by novel avian influenza A (H7N9) virus infection based on the mean value of lymphocyte counts, Jiangsu province, Eastern China, 2013 (n = 28).
(TIF)
