# Supplementary figures and images for: Epidemiological and Clinical Characteristics and Risk Factors for Death of Patients with Avian Influenza A H7N9 Virus Infection from Jiangsu Province, Eastern China
Source: PLoS One. 2014 Mar 4;9(3):e89581. doi: 10.1371/journal.pone.0089581 (PMC3942409; doi:10.1371/journal.pone.0089581)

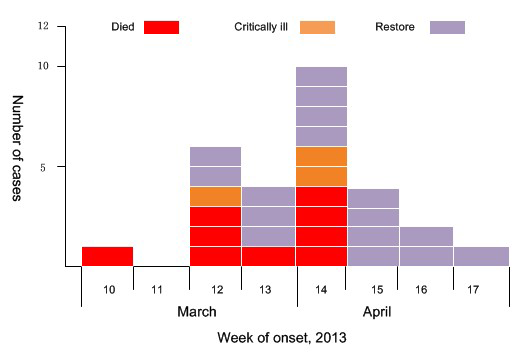

Supplement: Figure S1 — Onset time distribution (by week) of cases with avian influenza A (H7N9) virus infection, Jiangsu province, Eastern China, 2013 (n = 28). (TIF) [file pone.0089581.s001.tif]

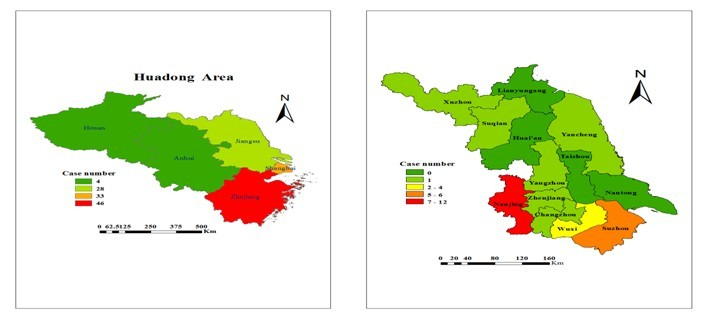

Supplement: Figure S2 — Geographical distribution of cases with a novel avian influenza A (H7N9) virus infection, Jiangsu Province, Eastern China, 2013 (n = 28). (TIF) [file pone.0089581.s002.tif]

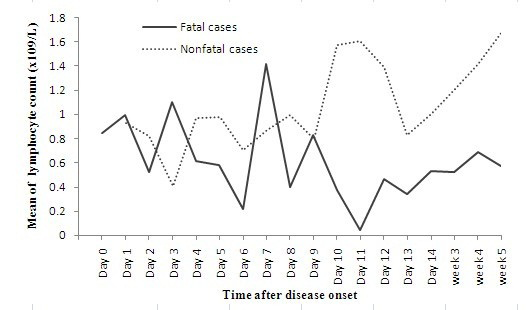

Supplement: Figure S3 — Stochastic natural cause of disease caused by novel avian influenza A (H7N9) virus infection based on the mean value of lymphocyte counts, Jiangsu province, Eastern China, 2013 (n = 28). (TIF) [file pone.0089581.s003.tif]
